# Sofosbuvir Plus Daclatasvir in Treatment of Chronic Hepatitis C Genotype 4 Infection in a Cohort of Egyptian Patients: An Experiment the Size of Egyptian Village

**DOI:** 10.1155/2018/9616234

**Published:** 2018-03-20

**Authors:** Ossama Ashraf Ahmed, Eslam Safwat, Mohamed Omar Khalifa, Ahmed I. Elshafie, Mohamed Hassan Ahmed Fouad, Mohamed Magdy Salama, Gina Gamal Naguib, Mohamed Mahmoud Eltabbakh, Ahmed Fouad Sherief, Sherief Abd-Elsalam

**Affiliations:** ^1^Internal Medicine Department, Gastroenterology and Hepatology Unit, Ain Shams University, Cairo, Egypt; ^2^Tropical Medicine Department, Ain Shams University, Cairo, Egypt; ^3^Tropical Medicine & Infectious Diseases Department, Tanta University, Tanta, Egypt

## Abstract

**Background and Aims:**

As indicated by the World Health Organization (WHO), Egypt is positioned as the country with the world's highest prevalence of Hepatitis C virus (HCV). HCV is transmitted through unexamined blood transfusions, different employments of syringes, and poor cleansing, as per the WHO. Our study aimed at screening and management of chronic hepatitis C genotype 4 infected patients in Bardeen village, Sharkeya Governorate, Egypt, with Sofosbuvir plus Daclatasvir, as well as estimating the safety and efficacy of that regimen.

**Methods:**

Screening of adult patients in Bardeen village was done from March 2016 till November 2016 using hepatitis C virus antibodies by third-generation ELISA testing. Positive results were confirmed by PCR. Patients eligible for treatment received Sofosbuvir 400 mg and Daclatasvir 60 mg daily for 12 weeks and were assessed for sustained virologic response at 12 weeks following the end of treatment (SVR 12).

**Results:**

Out of 2047 subjects screened for hepatitis C virus, 249 (12.2%) showed positive results. 221 out of those 249 subjects (88.7%) had detectable RNA by PCR. Treatment of eligible patients (183 patients) with Sofosbuvir plus Daclatasvir for 12 weeks resulted in 96% achievement of sustained virologic response at week 12. Adverse events were tolerable.

**Conclusion:**

Sofosbuvir plus Daclatasvir regimen is safe and effective for treatment of chronic hepatitis C Genotype 4 infected patients with minimal adverse events. HCV eradication program implemented in Egypt can be a model for other countries with HCV and limited resources. The availability of generic drugs in Egypt will help much in eradication of the virus.

## 1. Introduction

Several recent studies discussed the epidemiology of HCV infection worldwide. It is estimated that 10.4 million patients are infected with HCV genotype 4 which accounts for 13% of all HCV infections worldwide. HCV genotype 4 infection is common in the Middle East, Northern Africa, and Sub-Saharan Africa. In Egypt, 15% of an estimated population of 80 million is HCV positive, of which 93% are infected with genotype 4 [1–5].

During the past few years, there have been enormous efforts to understand the structure and life cycle of hepatitis C virus (HCV) and to develop specific medications targeting different viral proteins such as NS3/4A protease, NS5B polymerase, and NS5A replication complex. The discovery of direct acting antiviral agents (DAAs) represented a revolution in the management of chronic hepatitis C virus infection [6].

The pipeline of treatment regimens included combining interferon with DAAs with or without Ribavirin. Later on, interferon-free regimens were introduced through combining different classes of DAAs together. A need has arisen for a DAA based regimen that is pangenotypic, is well tolerated with minimal adverse effects, has low pill burden, and shows minimal drug interactions [7].

Daclatasvir is an NS5A inhibitor with pangenotypic activity that is effective against the six major HCV genotypes with a pharmacokinetic profile permitting once-daily dosing. Daclatasvir is well tolerated. Headache is the most frequently encountered adverse event. Daclatasvir is a weak inducer of cytochrome P450 and thus it has minimal drug interactions. In addition, its metabolism is mainly hepatic, which permits its use without dose adjustments in patients with chronic kidney disease. For prevention of emergence of resistant infections, an appropriate dose of Daclatasvir in combination with other suitable DAAs is recommended [8].

Sofosbuvir is another pangenotypic oral NS5B inhibitor with once-daily dosing that is both effective and tolerable. It has few drug interactions. Sofosbuvir/Daclatasvir combination is associated with a high rate of SVR in genotype 1 or 4 patients who are assumed to be difficult to treat. Adding Ribavirin increases the SVR rate in treatment-experienced and cirrhotic patients [4, 8].

Both agents have proven effectiveness against HCV genotype 4 infections in phase III ALLY-1, ALLY-2, and ALLY-3 studies that evaluate the role of this combination among different HCV genotypes. Phase II IMPACT study evaluates this combination in addition to Simeprevir in treatment of HCV genotypes 1 and 4 DAA-naïve patients with portal hypertension or decompensated cirrhosis. All patients achieved SVR12 regardless of the presence of baseline detectable resistance-associated mutations in 83% of patients [9].

Our study aimed at screening and management of chronic hepatitis C genotype 4 infected patients in Bardeen village, Sharkeya Governorate, Egypt, with Sofosbuvir plus Daclatasvir, as well as estimating the safety and efficacy of that regimen.

## 2. Subjects and Methods

This study was a prospective observational study conducted in Bardeen village (Sharkeya Governorate, Egypt) between March 2016 and November 2016. Adult patients (≥18 years) underwent HCV screening using HCV antibodies by third-generation ELISA. Those with positive HCV antibodies were submitted to clinical, laboratory, and imaging assessment prior to treatment.

Before treatment, all patients were subjected to a full medical history including history of previous treatment for HCV and any features of decompensated cirrhosis and a thorough clinical examination. Baseline laboratory studies were done including liver function tests, kidney function tests with estimation of glomerular filtration rate (eGFR), complete blood count, alpha fetoprotein, HBsAg, pregnancy test for females at child bearing age, and abdominal ultrasonography. Blood samples were obtained from all patients, centrifuged, and then stored at −20°C until measurements. A quantitative measurement serum load of HCV was performed by real-time PCR (Cobas Ampliprep/Taqman HCV Monitor version 2.0, with a detection limit of 15 IU/ml; Roche Diagnostic Systems, Pleasanton, California, USA), and it was repeated at the end of treatment, and 12 weeks after treatment to detect SVR 12. All included patients were assumed to have HCV genotype 4.

Treatment-experienced patients and patients with hepatitis B virus coinfection, advanced liver disease (decompensated cirrhosis) or HCC, eGFR < 30 ml/min, haemoglobin less than 10 g/dl, or platelet count less than 50,000/mm^3^ were referred for further pretreatment assessment and were excluded from the current study.

Treatment eligible patients received Sofosbuvir 400 mg and Daclatasvir 60 mg daily for 12 weeks. During treatment, they were closely monitored at week 2, week 4, week 8, and week 12 by laboratory studies including CBC, creatinine, AST, ALT, and total bilirubin.

The primary end point of our study was detecting the percentage of genotype 4 hepatitis C virus infected treatment-naïve participants with sustained virologic response at follow-up week 12 (SVR12). The secondary end point of our study was detection of any adverse events with treatment.

All patients signed a written informed consent prior to inclusion into this study and institutional ethical committee in Tanta University Faculty of Medicine approved the study. The study protocol conforms to the ethical guidelines of the 1975 Declaration of Helsinki (6th revision, 2008).

All collected data were analysed and correlated. Statistical analysis was performed using the Statistical Package for Social Sciences (SPSS) version 24. Basic descriptive statistics including means and standard deviations were performed. Comparison of qualitative data between groups was performed using Chi-square test. Independent *t*-test or Mann–Whitney *U* test were used to compare quantitative data between groups with parametric distribution or nonparametric distribution, respectively. The continuous variables across time were compared using paired *t*-test or Wilcoxon Signed-Rank Test. Differences were considered statistically significant if the *P* value was less than 0.05.

## 3. Results

This study was conducted between March 2016 and November 2016 in Bardeen Village from Sharkeya Governorate, Egypt. 2047 adult Egyptians were screened for HCV antibodies by ELISA. 249 subjects (12.2%) showed positive results. Real-time PCR for detection of HCV RNA was done for antibody positive patients where 28 subjects (11.3%) were found to have undetectable level of HCV RNA, whereas 221 subjects (88.7%) had detectable HCV RNA by PCR.

Further assessment of the 221 patients that had detectable PCR for HCV RNA yielded 183 patients (82.8%) that were eligible for treatment, while 38 patients (17.2%) were deferred from the study according to our exclusion criteria. 19 of those patients suffered advanced liver disease (decompensated cirrhosis and HCC) (50%), 3 patients were pregnant (7.9%), another 3 patients were coinfected with hepatitis B virus (7.9%), and 13 more patients had hematologic problems (platelet count below 50000/mm^3^ or haemoglobin level below 10 gm/dl) (34.2%%).

**Figure 1:**
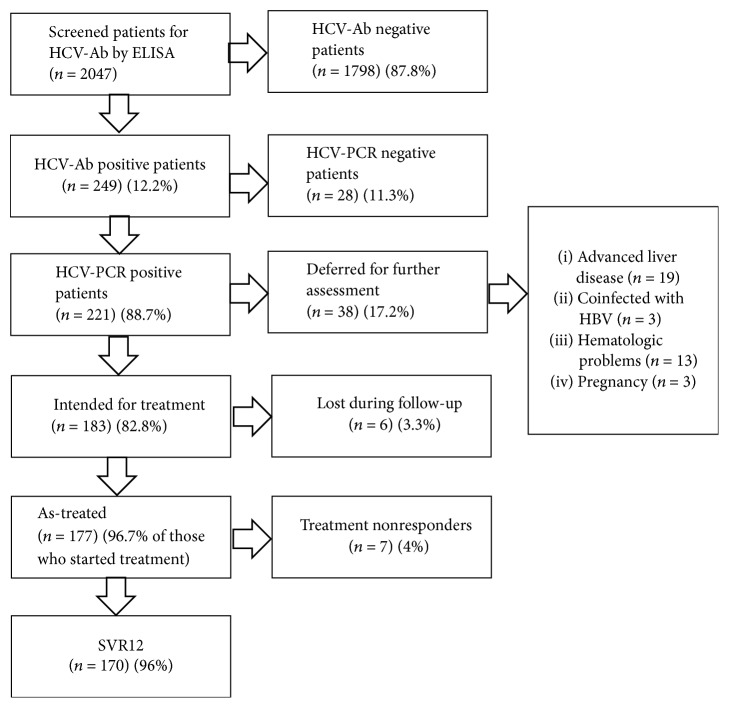
Study design.

**Figure 2:**
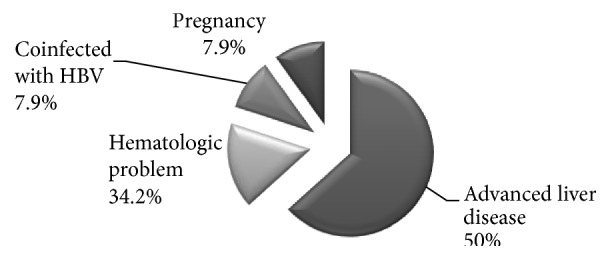
Analysis of causes of deferral from the current study.

Treatment eligible group included 183 patients who started Sofosbuvir 400 mg daily plus Daclatasvir 60 mg daily for 3 months. After starting treatment, 6 patients (3.3%) were lost during follow-up, while 177 patients (96.7%) completed their treatment course. Their mean age was 48.53 ± 12.93. 75 patients were males (42.4%) and 102 patients were females (57.6%) (Figures 1 and 2).

Neither age nor gender nor pretreatment biochemical profile showed any statistically significant correlation with treatment outcome (Table 1).

**Figure 3:**
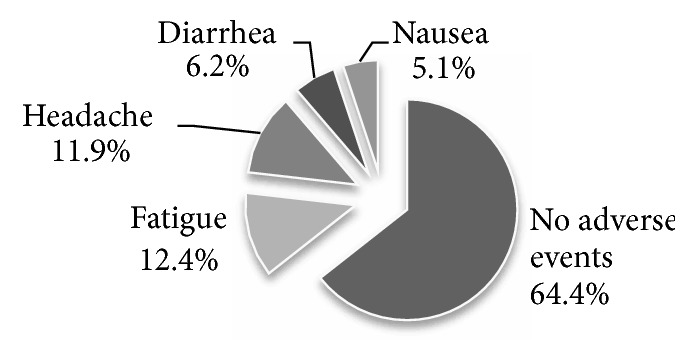
Adverse events for Sofosbuvir/Daclatasvir regimen.

114 of the as-treated group (64.4%) tolerated medications with no notable adverse events. 22 patients (12.4%) experienced fatigue, 21 patients (11.9%) experienced headache, 11 patients (6.2%) experienced diarrhea, and 9 patients (5.1%) experienced nausea (Figure 3).

Within the 177 patients who completed 12 weeks of treatment, 170 patients (96%) achieved SVR12 (negative PCR at week 12 following end of treatment). 7 patients (4%) were treatment nonresponders and were scheduled for retreatment in the nonresponders clinic.

Regarding treatment responders, pretreatment ALT showed a median value of 40 IU/L, while posttreatment ALT showed a median of 24 IU/L. Pretreatment AST showed a median value of 40 IU/L, while posttreatment AST showed a median value of 25 IU/L. Statistically significant reductions in ALT and AST were observed in responders following 12 weeks of treatment (*P* < 0.001).

## 4. Discussion

Chronic hepatitis C virus infection is a major health issue worldwide. It is the leading cause of liver-related morbidity and mortality in Egypt and one of the most common indications for liver transplantation. The introduction of direct acting antiviral agents shifted the management of chronic HCV infection to a new level. The combination of Sofosbuvir plus Daclatasvir proved to be very effective in treating hepatitis C virus infection, particularly genotype 1. However, data on genotype 4 are still deficient.

This observational study was conducted on a cohort of patients from Bardeen village, Sharkeya Governorate, Egypt. The prevalence of HCV antibody positive subjects and that of RNA positive subjects were 12.2% and 10.8%, respectively. This goes in agreement with CDC statistics which estimates the prevalence of chronic HCV infection in Egypt as 10%.

More than 2000 individuals were screened where 12% of them were anti-HCV positive. The majority of these were HCV RNA positive, and only 11% had spontaneously cleared their HCV infection. In the current study, 96% of as-treated population showed SVR12.

Only 177 of the screened patients were directly managed by our team. Only 7 of them were nonresponders. They were referred to “nonresponders clinic” where they started receiving Sofosbuvir/Daclatasvir/Simeprevir/Ribavirin regimen and were managed separately.

Sulkowski et al. studied the effect of Sofosbuvir plus Daclatasvir on treatment naïve genotype 1 chronic hepatitis C infected patients in one arm of their study which showed that 100% of patients in that arm (*N* = 41) achieved SVR12 after completing 12 weeks of treatment [10].

In a study by Fontaine et al. in 2015, 82 genotype 4 infected patients were treated with Sofosbuvir plus Daclatasvir with or without Ribavirin and with or without Simeprevir. The 33 patients who received Sofosbuvir plus Daclatasvir only were subjected to statistical analysis. SVR12 was achieved in 88.9% of those patients. However, this could be explained by the fact that the studied group included patients who were difficult to treat, whether because they were treatment-experienced or with advanced liver disease [11]. Another large study in Egypt documented the high SVR12 in patients receiving generic Sofosbuvir and Daclatasvir [12].

Regarding the patients who were excluded from the study, 19 patients with advanced liver disease were referred as part of our protocol to “Advanced Cirrhosis Clinic” where they receive assessment for liver transplantation and possible preemptive therapy. However, we did not include them in our study. Thirteen patients with hematological problems including anemia, thrombocytopenia, and other suspected blood disorders were routinely referred to “Hematology Clinic” where they were investigated for the cause of the hematologic disorder and then referred back for comanagement of hepatitis C in specialized HCV treatment unit with hematology. Three coinfected HBV/HCV patients were referred as part of our protocol to “Coinfection Clinic” in specialized hepatitis C treatment center where they had undergone further assessment for combined HBV/HCV treatment with Sofosbuvir/Daclatasvir/tenofovir. However, we did not include them in our study. Three pregnant ladies were listed for treatment after delivery. However, we did not include them in our study. This real life experiment describes the challenges which we face in our attempt for complete eradication of hepatitis C virus.

In the current study, 114 of the as-treated group (64.4%) tolerated Sofosbuvir plus Daclatasvir with no notable adverse events. None of our patients died during treatment and none of them stopped treatment due to significant adverse events. 22 patients of the as-treated population (12.4%) experienced fatigue, 21 patients (11.9%) experienced headache, 11 patients (6.2%) experienced diarrhea, and 9 patients (5.1%) experienced nausea. This came in accordance with Babatin et al. who studied 96 patients with chronic HCV genotype 4 who were treated by Sofosbuvir plus Daclatasvir or Sofosbuvir plus Simeprevir. The Sofosbuvir plus Daclatasvir arm included 40 patients. 42.5% of those patients developed adverse events where 27.5% suffered fatigue, 25% suffered headache, 7.5% suffered nausea, 10% suffered insomnia, 3% suffered nausea, and 4% suffered diarrhea [13].

There was significant reduction in AST and ALT level (*P* < 0.001 for both) after completion of the antiviral treatment which came in accordance with one previous study by Mehta et al. in 2017 who studied the safety and efficacy of Sofosbuvir and Daclatasvir for treatment of chronic hepatitis C infection in a group of patients with B-Thalassemia major. Treatment responders showed significant improvement in transaminases compared to pretreatment values after 12 weeks of treatment with Sofosbuvir plus Daclatasvir indicating their role in improving necroinflammation in patients with chronic HCV. However, the study was carried on genotype 3 thalassemic patients [14].

In the current study, neither age nor any of the pretreatment biochemical markers including haemoglobin, leukocytic count, platelet count, ALT, AST, albumin, INR, bilirubin, and PCR for HCV RNA showed significant correlation with treatment outcome. To our knowledge, this is one of the first studies to assess the pretreatment laboratory results as predictors for HCV genotype 4 response to Sofosbuvir plus Daclatasvir regimen.

A more interesting part is the epidemiology and the actual percentage of persons that were reached and treated in this study. The introduction of novel direct acting antiretroviral drugs in treating hepatitis C in Egypt has led to widespread treatment to become the largest program to treat hepatitis C virus worldwide [15]. Thanks to novel drugs, we are now talking of the possibility of eradicate hepatitis C virus. Also, the availability of generic drugs reduced costs, and will help in achieving complete eradication of HCV.

Finally, we can conclude that Sofosbuvir and Daclatasvir are effective in treatment of chronic HCV genotype 4 infections with minimal adverse events. Pretreatment liver chemistry does not seem to correlate with treatment outcome.

## Figures and Tables

**Table 1:** Relation between patients' age and baseline biochemical parameters and achievement of SVR.

Baseline data	Response to Treatment		*P*
	Nonresponder (*n* = 7) Mean ± SD/median (IQR)	Responder (*n* = 177) Mean ± SD/median (IQR)	
Age	48.86 ± 9.17	48.57 ± 13.09	0.95^*∗*^
ALT (IU/l)	24 (21–41)	40 (26.75–60.25)	0.2^*∗∗*^
AST (IU/l)	35 (20–56)	40 (27–67)	0.39^*∗∗*^
S. albumin (g/dl)	3.7 (3.3–4.2)	4.1 (3.87–4.4)	0.056^*∗∗*^
Total bil. (mg/dl)	0.7 (0.45–1)	0.7 (0.5–0.9)	0.69^*∗∗*^
INR	1.05 (1–1.19)	1.05 (1–1.13)	0.96^*∗∗*^
HCV-RNA	266591 (110000–3800000)	220500 (45000–864307.25)	0.95^*∗∗*^
TLC (×10^3^/mm^3^)	5.9 (4.5–8.3)	7 (4.85–8.35)	0.51^*∗∗*^
HGB (gm/l)	12.63 ± 0.64	12.94 ± 1.75	0.72^*∗*^
PLT (×10^3^/mm^3^)	186.29 ± 85.35	233.61 ± 66.91	0.07^*∗*^
Creatinine (mg/dl)	0.94 ± 0.21	0.87 ± 0.22	0.44^*∗*^

^*∗*^Independent  *t*-test; ^*∗∗*^Mann–Whitney *U* test; ALT: alanine transaminase, AST: aspartate transaminase, bil.: bilirubin, HGB: haemoglobin, INR: international normalized ratio, IQR: interquartile range, TLC: total leucocytic count, PLT: platelet.
